# Development of a DNA aptamer to detect *Brucella abortus* and *Brucella melitensis* through cell SELEX

**Published:** 2020

**Authors:** Z. Nosaz, S. Rasoulinejad, S. L. Mousavi Gargari

**Affiliations:** 1 MSc in Microbial Biotechnology, Department of Biology, Faculty of Basic Sciences, Shahed University, Tehran, Iran;; 2 Max Planck Institute for Polymer Research, Ackermannweg 10, 55128, Mainz, Germany;; 3 Department of Biology, Faculty of Basic Sciences, Shahed University, Tehran, Iran; ** These authors contributed equally to this work

**Keywords:** Brucella, Cell SELEX, DNA aptamer, Flow cytometry

## Abstract

**Background:**

Brucellosis is a zoonosis, caused by *Brucella* spp. which are small aerobic intracellular coccobacilli, localized in the reproductive organs of host animals, causing abortion and sterility. The diagnosis of this zoonosis is based on microbiological, serological or real time-polymerase chain reaction (RT-PCR) laboratory tests. Although the common microbiological and serological based assays have advantages, they are not able to solve the diagnosis problems.

**Aims:**

To overcome some of the limitations of present techniques, in this study, we developed an aptamer through whole-cell systematic evolution of Ligands by EXponential enrichment (SELEX) procedures to detect *Brucella*.

**Methods:**

We used mixture of *Brucella melitensis* and *Brucella abortus* as the target. In order to prepare the single-stranded DNA (ssDNA) aptamer, the DNA library was amplified with 5´-phosphorylated reverse primer and treated with lambda exonuclease. The SELEX procedure was performed by incubating the ssDNA pool with a bacterial suspension in a binding buffer. The selected procedures were monitored by flow cytometry using FITC-labelled forward primer. Aptamers with the highest binding affinity towards the target and the lowest to other strains were selected.

**Results:**

Two aptamers namely B20 and B21 showed significant binding affinity toward *B. melitensis* and *B. abortus*. The dissociation constant (K_d_) for aptamers B20 and B21 was 40.179 ± 3.06 pM and 184.396 ± 465 pM, respectively.

**Conclusion:**

The isolated aptamers were able to identify *B. melitensis* and *B. abortus* with a remarkable binding efficiency and appropriated K_d_ in a picoMolar range and therefore can be good candidates in the development of any rapid assay test implanted on routine brucellosis diagnoses.

## Introduction

 Brucellosis is a common human and animal disease affecting a wide range of livestock and all age groups. Brucellosis is still counted as a major general hygiene concern in the world and is common as a zoonosis. The major clinical manifestations of brucellosis in animals can be found as arthritis, mastitis and lameness, abortion and inflammation of the epididymis (Abebe et al., 2010 ▶). In humans, *Brucella melitenis* is the most known acute disease and *Brucella abortus* can create subacute forms (Peterson et al., 2015 ▶). In Iran, brucellosis is an endemic, severe health issue that has increased recently (Shakerian and Nodargah, 2018 ▶). Annual cases of brucellosis are reported to be about 27500, mostly caused by *B.* *melitenis* (Mostafavi and Asmand, 2012 ▶). In humans, it causes infectious or noninfectious manifestations, which make the early diagnosis difficult (Lucero et al., 2008 ▶). Bacteriological, serological, and molecular assays are the three common methods in the diagnosis of brucellosis infections. In most of these common assays, isolating the causative agent by culture is necessary. Although diagnosis based on bacteriology counts as a standard test, prolongs cultivation periods which at least needs 4-5 days for detection; also, difficulties of bacterial isolation and unsuccessful culturing are the worst drawbacks of common methods (Mousa et al., 1988 ▶). In serological assays, body fluid and antibodies are used as a common approach. The possibility of cross reactivity with other pathogens makes the results of serological assays less trustworthy. Although antibodies play an important role in detection and diagnosis, low stability and high production costs are some of the limitations of antibody-based detections. The tests are based on lipopolysaccharide (LPS) detection and because of the low specificity of the immunodominant epitope of *Brucella* and its similarity to other equivalent epitopes in *Escherichia coli*, *Salmonella* (sp), *Y*e*rsinia* (sp), *Vibrio* (sp), and some other spices, they cannot count as accurate assays. Molecular methods like polymerase chain reaction (PCR) and real time-PCR (RT-PCR) could somehow overcome the aforementioned drawbacks of serological and culture based diagnostic methods but still in some cases sensitivity and specificity are not 

satisfying (Ducrotoy et al., 2018 ▶). Due to the small amount of *Brucella* present in clinical samples, the direct detection of DNA in brucellosis is another challenging issue. Ultimately, the efficiency of the molecular detection can be affected by the quality of the extracted DNA and the type of clinical samples (Minda and Gezahegne, 2016 ▶; Ducrotoy et al., 2018 ▶; Shakerian and Nodargah, 2018 ▶). Since brucellosis is a zoonosis, developing an accurate, fast, efficient and cost effective assay will be useful, especially in rural life, due to the difficulty in accessing advanced equipment. Aptamers are short, single-stranded oligonucleotides with a length between 60-100 bp (Tsao et al., 2017 ▶). The single stranded molecule can fold and twist into spatial structures and specifically bind to the target. The dissociation constant (K_d_) of aptamers toward their targets is ranged from micro to picoMolar (Gewirtz, 1999 ▶; Brody and Gold, 2000 ▶). Their target can differ from small molecules to large macromolecules such as large proteins and even whole cells. In 2017, the Food and Drug Administration approved the therapeutic application of six aptamers (Volk and Lokesh, 2017 ▶; Ladju et al., 2018 ▶). To date, numerous aptamer sequences have been characterized with different purposes in diagnostic and therapeutic fields (Blind and Blank, 2015 ▶; Chen et al., 2017 ▶; Dunn et al., 2017 ▶; Zhou and Rossi, 2017 ▶). Furthermore, the combination of aptamers with some chemicals can help enhance their activity or stability (Ladju et al., 2018 ▶). The binding affinity of an aptamer can be enhanced by engineering their sequences (Volk and Lokesh, 2017 ▶). The backbones of aptamers are flexible enough to conjugate with drugs, nanoparticles with functional groups, providing an adjustable approach for detection (Gopinath et al., 2014 ▶; Fakhri et al., 2018 ▶; Morita et al., 2018 ▶).

 In order to overcome some of the aforementioned limitations, we developed an aptamer through a whole cell systematic evolution of Ligands by EXponential enrichment (SELEX) procedure to detect *Brucella*. We used *B. melitensis* and *B. abortus* as the mixture target. Using a mixture of bacteria as target provides a wide detection range toward *B. melitensis* and *B. abortus*. The selected aptamer can be used in any tool designed for the detection of *B. melitensis* and *B. abortus*.

## Materials and Methods

**Bacterial strains preparation**

 All bacterial species used in this study are listed in Table 1. All strains were cultured in Luria-Bertani broth (LB) and maintained in LB agar, except *Haemophilus influenza*, *Streptococcus pneumonia*, and *Neisseria meningitidis* type B, cultured in 5% defibrinated sheep’s blood agar in aerobic conditions at 37°C. Cells were cultured and counted using a standard plate counting method. *Brucella melitensis* and *B. abortus* (OD_600_=0.4) were mixed in a suspension with a 1:1 ratio. A total cell density of 10^8^ cell/ml of this mixture was centrifuged at 1000 × g for 10 min. Cells were washed 3 times with phosphate buffer saline 1X, 0.02% Twee-20 (PBST). The supernatant was discarded and the pellet was suspended in 300 µL of binding buffer (PBST plus 1% bovine serum albumin) to be used in the SELEX procedure. Likewise, other strains, for counter SELEX, were prepared in a suspension with a 1:1 ratio (10^8^ cell/ml) l ml in a total volume of 10 ml. One ml of homogeneous cell suspension was centrifuged, washed 3 times with PBST and resuspended in 300 µL of PBS. *Escherichia coli* TOP10 competent cells were used for the transformation of the enriched library.

**DNA library amplification**

 The DNA library and primers were purchased from Metabion (Steinkirchen, Germany). The initial library (10 µM) was amplified by PCR using the following primers:

Forward: 5´-GCCTGTTGTGAGCCTCCTAAC-3´

Reverse: 5´-GGGAGACAAGAATAAGCA-3´

 The amplification was performed at 95°C for 5 min as an initial denaturation, then 30 cycles of 94°C denaturation, 62°C annealing, and 72°C extension all for 30 s, and 72°C for 5 min as the final extension. All PCR products were checked on 1.5% agarose in Tris-borate-EDTA buffer (TBE 0.5X) and were purified with ethanol precipitation assay. The purified DNA concentration was measured by Nano drop (Pico200 Spectrophotometer, UK). Subsequent purification of the PCR products used in SELEX procedures was carried by gel purification kits according to the manufacturer’s protocol.

**Table 1:** Bacterial species used in this study

**SELEX target**	Collection center
*Brucella melitensis* 16M ATCC 11649	Razi Vaccine and Serum Research Institute
*Brucella abortus* 544 ATCC 21749	
**Counter SELEX and Cross test**	
*Vibrio cholorae* clinical strain	Shahed University
*Pseudomonas aeruginosa* ATCC 27853	
*Shigella sonnei* clinical isolated	
*Listeria monocytogenes* ATCC 7644	
*Staphylococcus aureus* ATCC 25923	
*Acinetobacter baumannii* ATCC 19606	
*Escherichia coli* ATCC 25922	
*Haemophilus influenza* type b ATCC 10211	
*Streptococcus pneumonia* ATCC 33400	
*Neisseria meningitidis* type B ATCC 13090	
*Salmonella typhi* ATCC 700931	
*Yersinia enterocolitica* ATCC BAA-1511D-5	
**Competent cell**	
*Escherichia* * coli* TOP10	

**Single stranded DNA (ssDNA) library preparation**

 The DNA library was amplified with 5´-phosphorylated reverse primer in order to prepare the ssDNA library. The purified 5´-phosphorylated dsDNA was incubated at 37°C with 5 units of lambda exonuclease in a total volume of 20 µL reaction for 25 min. The reaction was terminated at 80°C for 15 min followed by 5 min incubation on ice.

**SELEX and counter SELEX processes**

 The SELEX procedure was performed as described previously (Rasoulinejad and Gargari, 2016 ▶). The ssDNA library (2 nM) was incubated at 95°C for 10 min, then transferred into an ice bath. The ssDNA pool was added to 300 µL of a suspension of *B. melitensis* and *B. abortus* bacteria, in a binding buffer and incubated for 1 h at room temperature with gentle shaking. The mixture was centrifuged at 1000 × g for 10 min. The supernatant was removed and the cells were washed with 1 ml of the wash buffer (PBST). Incubation time was gradually reduced from 60 min to 10 min and washing times were increased from 1 to 10 times from 1st to 14th SELEX rounds. In order to recover the binding aptamers, the cells were resuspended in 100 µL of miliQ water, heated at 95°C for 10 min and centrifuged at 5000 × g for 30 s. The recovered ssDNA was amplified with PCR and used for the next round of SELEX. In total, fourteen rounds of SELEXs were carried out and the selection processes were monitored by flow cytometry.

**Counter SELEX**

 The 3rd, 6th, 7th and 11th rounds were proceeded as counter SELEX. Each counter SELEX included two steps; a negative selection followed by positive selection. The ssDNA pool obtained from previous rounds of SELEX was added to the 300 µL suspension of all counter strains in the binding buffer. This mixture was incubated for 60 min at room temperature with gentle shaking. Cells were precipitated and the supernatant was transferred to another tube and used as a source of aptamer for further SELEX rounds.

**Enrichment of ssDNA aptamers pool**

 In order to monitor the selection procedures, the outcome of 2nd, 5th, 11th, 13th and 14th SELEXs were used as templates for PCR using forward FITC labelled primer. The assay was carried out by incubating 50 pM of ssDNA from each round with a mixture of *B. melitensis* and *B. abortus* at room temperature for 60 min. Cells were collected and washed with the wash buffer. The pellet of cells and bound ssDNA aptamers was resuspended with 1 ml of binding buffer. The percentage of fluorescent intensity of bound ssDNA aptamers was measured by flow cytometry.

**Maintenance of obtained ssDNA pool**

 The recovered outcome of the 14th round of SELEX was amplified with the PCR. In order to introduce the 3´ “A” overhang added by Taq DNA polymerase, the final extension at 72°C was carried out for 10 min. The PCR product was cloned on a pTZ57R/T vector and transformed to *E. coli* TOP10 competent cells. Colonies were tested using colony PCR with free-labelled primers. Positive clones were kept in glycerol and saved at -80°C for further studies.

**Affinity selection**

 Each positive clone was used as a template to amplify aptamers with FITC-labelled primer. The labeled aptamers, made as ssDNA then were incubated with *B. melitensis* and *B. abortus*. The percentage of binding affinity of each aptamer was measured. The DNA aptamers with highest binding affinity were selected for counter- and cross-tests. The counter-test was performed by incubating counter bacterial strains with high binding aptamers toward the target. Four aptamers with the highest binding affinities toward the target were selected for a cross-reactivity test. All four aptamers were incubated individually with *E. coli*, *Salmonella typhi*, *Yersinia* *enterocolitica*, and *Vibrio cholerae*. Finally, the aptamers with the highest binding affinity toward the target and the lowest towards other strains were selected for further investigation.

*Measurement of* *dissociation constant*

 Determination of K_d_ was carried out by the procedure described previous by (Alfavian et al., 2017 ▶). In brief, different concentrations of FITC labeled aptamer (0-200 pM) were incubated with fixed concentrations of bacterial cocktail (*B. melitensis* and *B. abortus*) at room temperature for 45 min. The bacteria pellets were collected, washed with the washing buffer and suspended in 1 ml binding buffer. The fluorescent intensity of each aptamer concentration was measured by flow cytometry. The K_d_ was calculated and the curve was plotted as follows:

Y = BmaxX / (K_d_ + X) equation

(Jandel, San Rafael, CA, USA) by Sigmaplot 12.0.

**Sequencing and secondary structure prediction**

 The selected aptamers, with the highest affinity toward *B. melitensis* and *B. abortus* and the lowest toward other strains was sent for sequencing (Bioneer, Daejeon, South Korea). The alignments were performed by CLC bio workbench (CLC) sequence alignment 7.8.1 software. The secondary structures of aptamers was analyzed by the Zuker algorithm, using Oligo Analyzer 3.1 web server. The prediction analysis was carried out under 144 mM Na^+^ and 1 mM mg^+2^ ion conditions at room temperature.

## Results

**DNA aptamer pool and SELEX Procedures**

**Fig. 1:**
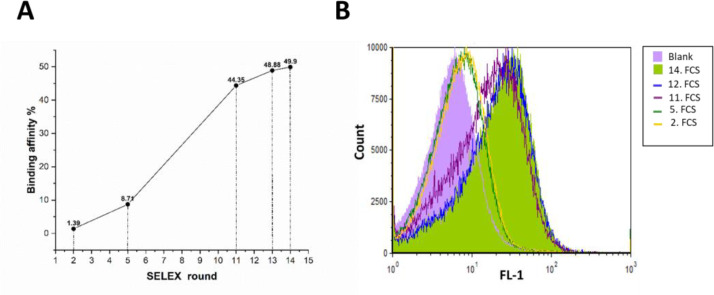
Binding affinity toward target cells through SELEX procedures. (**A**) Development of Aptamer pool from beginning to end, and (**B**) Improvement of flow cytometry data through SELEX. The numbers in the legend referring to each SELEX cycle

 The PCR amplification reactions after each selection round were optimized. Factors influencing the results of the PCR reactions were concentration templates, MgCl_2_, and annealing temperature. The binding affinity of aptamer pools toward *B. melitensis* and *B. abortus*, measured by flow cytometry, showed significant increase at round 5, and the increscent continued to the 11th round of SELEX. The percentage binding affinity of rounds 2nd, 5th, 11th, 13th and 14th is demonstrated in Fig. 1A. The comparison of binding affinity between SELEX 13th and 14th indicated that the SELEX process can be ended in the 14th selection round (Figs. 1A and B).

**Transformation**

 The aptamers obtained from the 14th selection round were cloned in the pTZ57R/T vector and transformed to *E. coli* TOP10 competent cells. The results of colony PCR showed 24 positive clones analyzed using agarose electrophoresis.

**Highest binding affinity selection**

 Nine out of 24 colonies showed the highest binding affinity toward *B. melitensis* and *B. abortus*. Two aptamers, namely B20 and B21, showed significant binding affinity toward *B. melitensis* and *B. abortus* compared to the initial library.

**Fig. 2:**
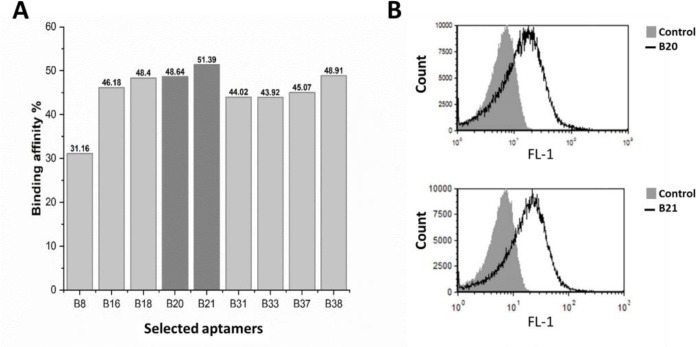
Representation of aptamers’ binding affinity. (**A**) Binding affinity of isolated aptamers toward *B. melitensis* and *B. abortus*, and (**B**) Selected flow cytometry result of isolated aptamer, B21 and B20

**Fig. 3:**
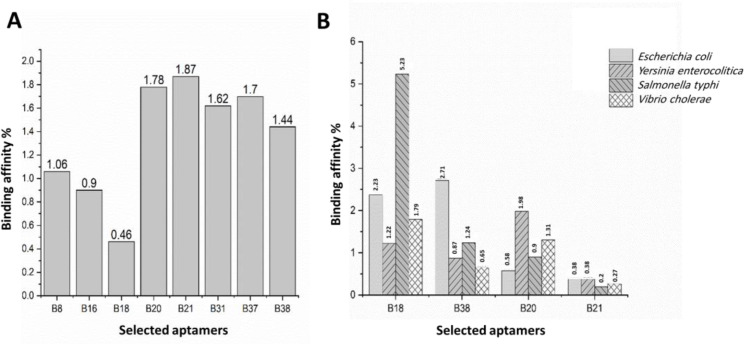
Binding affinity and selectivity of aptamer candidates. Each chart illustrates, (**A**) Counter, and (**B**) Cross reactivity, representing the binding affinity of aptamer candidates toward other competitor targets

**Fig. 4:**
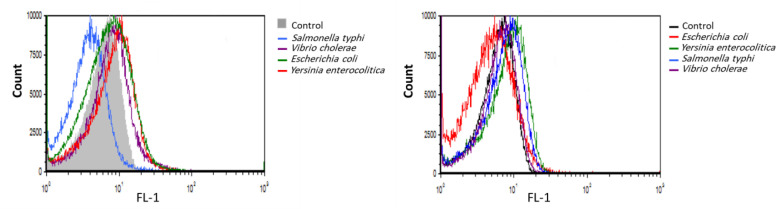
Flow cytometry of B20 and B21 against selected bacteria for cross-test. The measurement of flow cytometry indicated that B20 and B21 had moderate but non-significant affinity toward the bacteria that always cause false positives in common serological tests. The consequence of counter- and cross-reactivity tests shows the high specificity of the isolated aptamers. The results refer to B20 and B21 from left to right

 Out of 24 selected aptamers, the average binding affinity of 9 aptamers toward counter bacteria were less than 2%, where the binding affinity of B21 toward *B. melitensis* and *B. abortus* was about 51% (Figs. 2A and B). Based on the binding affinity to *B. melitensis* and *B. abortus* and counter cocktail (Fig. 2A), 4 aptamer were selected for cross reactivity test. Aptamer B20 and B21 demonstrated the lowest binding affinity to bacterial species selected for the cross-reactivity test (Figs. 3A-B) and therefore these 2 aptamers were selected as high binders for further studies. The result of the cross test is shown in Fig. 4.

**Fig. 5:**
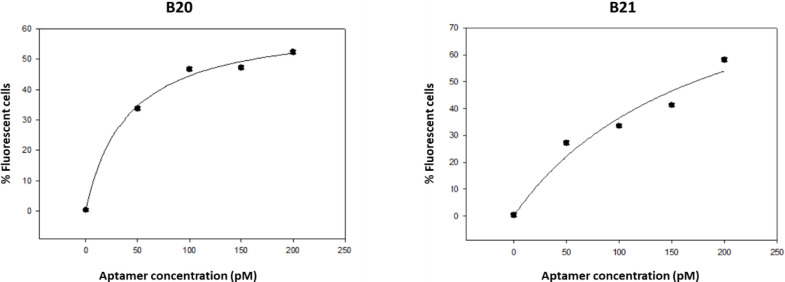
Dissociation constant curve. Using the binding affinity of each point concentration and ligand binding one site saturation equation with the calculated K_d_

**Dissociation constant calculation**

 The binding affinity of B20 and B21 aptamers toward *B. melitensis* and *B. abortus* was measured using different concentrations of aptamers (0, 50, 100, 150, and 200 pM). The calculated K_d_ for B20 and B21 were 40.179 ± 3.06 pM and 184.396 ± 465 pM, respectively (Fig. 5).

## Discussion

*Brucella* species as a zoonotic pathogen can be transmitted from animals such as sheep, goats, and cattle, 

resulting in brucellosis. This disease, as a common zoonotic infection, can cause significant endemic damage to the livestock industry, hence it is considered as an obstacle in the trade of livestock and their products. Unfortunately, *Brucella* does not show specific clinical symptoms making the diagnosis difficult. The development of an accurate, quick, easy and, low cost method is necessary for the detection and diagnosis of this disease. Recent fluorescent polarization assays (FPA) are promising methods that have paved the way to overcoming some of the previous problems encountered with *B. melitensis* and *B. abortus* detection. More studies are needed to prove the reproducibility of the assay. The real time-polymerase chain reaction has been used for the detection of *B. melitensis* and *B. abortus* as a rapid method. However, the genomic analysis of different *Brucella* species revealed high conservation between them, which makes designing species-specific RT-PCR assays difficult. Even probes sometimes result in a signal for all *Brucella* as well as non-*melitensis* species (Kaden et al., 2017 ▶). In addition, PCR-based methods require accurate and expensive instruments and professional operators (Kaden et al., 2017 ▶).

 The immunological method is also widely used for the detection of *Brucella.* Although this method is quick, shows cross-reactivity (Song *et al*., 2017). The 19KD antibody against outer membrane protein, used for the detection of brucellosis, did not show high sensitivity and specificity compared to PCR (Islam et al., 2018 ▶). In addition, S-LPS antigen is not present in all *Brucella* spp. and in the majority, false negative results are reported due to the presence of a single subgroup of immunoglobulin (Al Dahouk et al., 2013 ▶; Christoforidou et al., 2018 ▶).

 Due to their special folding and 3D structure, aptamers can specifically bind toward their targets and are compatible with antibody-target interactions, hence being good candidates to replace with antibodies. *Brucella melitensis* and *B. abortus* are two of the important species that cause brucellosis, and therefore detecting these two in single assays can reduce expenses as well as the time for detection. The characterization of aptamers B20 and B21 to prevent *B. melitensis* and *B*. *abortus* demonstrated a high binding affinity according to flow cytometry data and low K_d_ of 40.179 ± 3.06 pM and 184.396 ± 465 pM, respectively. The finding is consistent with our previous reports and comparable to other aptamers assigned by different targets through whole cell SELEX (Hamula et al., 2011 ▶; Bitaraf et al., 2016 ▶; Rasoulinejad and Gargari, 2016 ▶; Alfavian et al., 2017 ▶; Mirzakhani et al., 2018 ▶; Yu et al., 2018 ▶). Our findings are also in accordance with Shiply et al.’s (2010) ▶ study, where the researchers selected an aptamer against *E. coli* with a K_d_ of 96.98 pmol/L estimated by flow cytometry. Specific aptamers have also been selected for eukaryotic cell lines. Sgc8 aptamer with a picomolar K_d_ range was selected toward Hela cells (Chen et al., 2009 ▶). Similar research was conducted by Almasi et al. (2016) ▶ for LnCap cells. In addition, the high specificity of aptamers B21 and B20 can be attributed to the accuracy of cell SELEX as well as a dual selection performed in counter SELEX steps. To eliminate non-specific and weak binders, ascending stringency through selection rounds could also play a key role to access highly efficient aptamers. In cell SELEX, the aptamers are selected against whole live cells as a target. In whole cell SELEX, aptamers bind to cell surface proteins in their physiological condition, and we do not need to have information about the target toward which the aptamer is developed. However, in the cell SELEX method, aptamers do not bind to their target with full efficiency. This is because the expression levels of cell surface proteins in the bacterial pool are different (Dwivedi et al., 2013 ▶). In protein SELEX, aptamers bind to their target with a high specificity of nearly 100%, but there are several issues concerning protein SELEX. The aptamers may bind to domains of the target(s) which are not exposed to physiological conditions, especially in the case of membrane proteins. Moreover, the cost of protein purification is prohibitive and has its own problems. Aptamers B20 and B21 showed the highest specificity toward *B. melitensis* and *B. abortus* without having a significant affinity for counter-cells and other bacteria which affect the false positive results in serological tests. Counter-SELEX is a strategy to eliminate nonspecific bindings when an aptamer encounters a large amount of non-target proteins on a cell surface. The combination of a positive selection and counter-SELEX promote the developmental procedure and reduce the cross-reactivity of selected aptamers to structures that are closely related to that of the main target. Previous reports indicate that cell-SELEX can increase the selection rounds without applying counter selection (Ohuchi, 2012 ▶; Shangguan et al., 2015 ▶). Data obtained by flow cytometry after counter SELEX was performed at the 3rd, 6th, 7th, and 11th rounds of selection showed a significant increase in the binding affinity with a steep slope. Therefore, we can assume that the selected aptamers are able to detect the *Brucella* family, specifically *melitensis* and *abortus*.

 In conclusion, the aptamers we developed were able to identify *B. melitensis* and *B. abortus* with a remarkable binding efficiency and appropriated K_d_ in a picoMolar range. Therefore, the isolated aptamers herein can be good candidates for further studies on the development of any rapid assay test implanted on routine brucellosis diagnosis.
